# Dual Crosslinked Collagen/Chitosan Film for Potential Biomedical Applications

**DOI:** 10.3390/polym11122094

**Published:** 2019-12-14

**Authors:** Rushita Shah, Pavel Stodulka, Katerina Skopalova, Petr Saha

**Affiliations:** 1 Centre of Polymer Systems, University Institute, Tomas Bata University in Zlin, Tř. T. Bati 5678, 760 01 Zlín, Czech Republic; skopalova@utb.cz (K.S.); saha@utb.cz (P.S.); 2 Gemini Eye Clinic, U Gemini 360, 760 01 Zlín, Czech Republic; stodulka@lasik.cz

**Keywords:** chitosan, collagen, dual crosslinking, polymeric biomaterial

## Abstract

The application of polymeric biomaterial scaffolds utilizing crosslinking strategy has become an effective approach in these days. In the present study, the development and characterization of collagen–chitosan hydrogel film has been reported on using dual crosslinking agent’s, i.e., tannic acid and genipin simultaneously. Incorporation of genipin imparts a greenish-blue color to the polymeric film. The effect of dual crosslinking and their successful interaction within the matrix was evaluated by infrared analysis spectroscopy. The porosity of the film was examined using scanning electron microscopy (SEM). Results of TGA determine the intermediate thermal degradation. Further, the crosslinking phenomenon has found primary impact on the strength of the films. Enzymatic degradation for the films was performed with lysozyme and lipase. The cell adhesion and proliferation was also accomplished using mouse embryonic cell lines wherein the cells cultured on the dual crosslinked film. The thriving utilization of such dual crosslinked polymeric film finds their applications in ophthalmology especially as an implant for temporary injured cornea and skin tissue regeneration.

## 1. Introduction

Regenerative medicine is a broad field that includes tissue engineering, which has a promising and developing approach with a great potential of replacement and rejuvenate any injured or diseased tissues [1]. It focuses also on preparing bio interactive scaffolds that mimic extracellular matrix of the body as well as treats the complex, often chronic diseases. Hence, it has gained much interest among the researchers from different disciplines such as material science, chemistry and medicine [1]. Tissue engineering materials are destined to react with the living body, so there are many conditions associated with selecting the materials [2]. The matrix for tissue supporting should exhibit high porosity, appropriate mechanical and physicochemical properties [3]. Hence, polymeric materials are preferred by the researchers. Several synthetic polymers like poly(lactic acid), poly(a-hydroxyesters) and poly(l-glycolic acid) are widely utilized in tissue engineering because of their biodegradable nature [2]. However, there are some drawbacks associated with the use of synthetic based polymers, i.e., the intermediate product obtained during non-enzymatic hydrolysis and the decrease of the local pH, which ultimately give rise to inflammatory reactions and harm the cells surrounding the implant site [4]. Further, it also reduces the polymers melting point as well as its degradation rate. To overcome this problem, biomaterial constructed utilizing natural origin based biopolymers like collagen [1], gelatin, chitosan [2], hyaluronic acid [5] and alginate [6] with improved biological activity, cell adhesion and proliferation are utilized as safe materials for tissue engineering. 

Collagen and chitosan are the most abundant biopolymers found in nature and exhibit a large spectrum of application in the biomedical field of science [7]. Collagen is a group of naturally occurring protein and is the mostly preferred biomaterial used in tissue engineering due to its excellent biocompatibility and lower antigenicity [8]. It is the main component of the connective tissue and proteins in mammals, overall constituting up to 25%–35% of the whole body protein content [9]. Collagens from bovine and pig skins are the main industrial source and utilized in functional foods, cosmetics and biomedical materials [10]. However, the major drawback dealing with homopolymer, collagen-based biomaterial is the rapid degradation and poor mechanical properties, which creates hindrance in several tissue engineering applications [11]. Thus, it becomes essential for blending of collagen with other polymers. This will enhance superiority, processability and performance of the materials generated thereafter. Chitosan, a polyatomic polysaccharide is present in soft bodied insects, crustaceans, bone plate of cuttlefish and squids [12,13]. It is synthesized through deacetylation reaction using chitin as raw material and it is a co-polymer of D-glucosamine and *N*-acetyl-D-glucosamine [12]. The molecular weight and degree of deacetylation of chitosan can be easily modified [14]. Chitosan is well-known for its biocompatibility allowing its use in topical ocular application, implantation, drug delivery, wound healing, hemodialysis membrane, tissue engineering, etc. [15,16,17]. It is bio-adhesive in nature because of its positive charge at physiological pH [18]. Pure chitosan possesses properties such as non-toxicity, biodegradable, antifungal, antibacterial as well as biological inertness and stability. Until now, it has been utilized in form of hydrogels, fiber membranes, beads, blends and porous scaffolds for several biological and clinical purposes [14].

In tissue engineering, the crosslinking phenomenon is reflecting more concern due to excellent stability among the polymeric blends and decreasing reactivity [19]. Crosslinking results in elasticity, decreasing solubility and viscosity of the polymer, gives strength and toughness to the biomaterial developed [20]. Crosslinking can be physical for, e.g., UV-radiation, microwave and dihydrothermal treatment [21]. Even though these methods can avoid the introduction of potential toxic residue, they fail to yield an increasing crosslinking degree [11]. Chemical crosslinkers commonly used are aldehydes (for e.g., glutaraldehyde, formaldehyde), carbodimides, polyepoxy compounds, etc. The main limitation concerned with chemical crosslinkers is the unreacted crosslinkers inside the scaffolds, which ultimately gives the risk of the formation of toxic products and also limited mechanical strength [21]. To overcome this issue, natural alternatives like tannic acid, genipin, citric acid, proanthocyanidin and ferulic acid are preferred.

Tannic acid is a natural plant based polyphenol compound that has diverse biological functions such as antiviral, anti-inflammatory, antioxidant and antimicrobial properties [22]. It can interact readily with biopolymers like collagen, chitosan, albumin and gelatin through non-covalent interactions like H_2_-bonding and hydrophobic effects [23,24]. Sionkowska A and group for the first time utilized tannic acid as crosslinker to modify the properties of chitosan-collagen mixture [7]. Genipin is an aglycone of geniposide comprising of a dihydropyran ring and an ester group, which is derived from the fruits of Gardenia Jasminoides Ellis. Traditionally, it is used to treat pyrogenic infection, febrile disease, sprain, swelling, etc. [25,26,27]. Genipin is materializing with a number of polymeric materials comprising primary amino groups for, e.g., chitosan, certain peptides, polypeptides by crosslinked covalent grid and gives blue colored fluorescent [21,27]. Due to its lowered toxicity, genipin has gained increasing interest in the field of biomaterial processing technique [28,29]. With respect to the prospective results, it is possible to develop genipin crosslinked biomaterial for ocular therapeutics, tissue repair and pharmacology [29,30]. There are several examples for the approach to utilize genipin ophthalmology or tissue engineering such as the Mi FL et al. group found out that genipin not only exhibits decreasing cytotoxicity as compared to glutaraldehyde and epoxy compounds but is also able to efficiently crosslink cellular tissues and biomaterials comprising of free amino groups [31]. The genipin cross-linked chitosan thin membrane improved the preservation of corneal endothelial cell density as well as showed anti-inflammatory activity, which was reported by Jui-Yang Lai [29]. Maria Grolik et al. reported genipin cross-linked chitosan-collagen blends for corneal tissue engineering [32]. Long Bi also used genipin cross-linked chitosan-collagen for cartilage regeneration [33]. 

The present research portrayed for the first time preparation of collagen–chitosan hydrogel film utilizing two different natural crosslinkers, i.e., tannic acid and genipin simultaneously. At the moment, there is also no such information reported hence, emphasis is put on fabrication of collagen/chitosan-based biomaterial that should persist an inert effect on the human body, moreover it is user friendly. The properties of the newly formed film were thoroughly studied in the form of its physical appearance, physico-chemical structure, swelling behavior, thermal analysis, mechanical properties, biodegradation and cell (mouse embryonic cell lines) adhesion studies. This kind of film will find potential application in ophthalmology especially wherein corneal epithelium is injured externally, skin tissue engineering, wound dressing and/or cartilage/tissue regeneration. 

## 2. Materials and Methods 

### 2.1. Materials 

Chitosan (from crab shell, highly viscous, viscosity >400 mPa·s, catalog number-9012-76-4), collagen (from bovine, catalog number-48165), phosphate buffer saline (sterile liquid, D8662), tannic acid (catalog number-403040) and genipin (catalog number-G4796) were obtained from Sigma-Aldrich, Prague, Czech Republic. Whereas acetic acid (99.8%, catalog number 19990-11000), sodium hydroxide (catalog number-15740-31000) and dimethyl sulfoxide (DMSO, catalog number 12630-11000) from Penta, Prague, Czech Republic. 

### 2.2. Preparation of Dual Crosslinked Collagen–Chitosan Film

**Figure 1:**
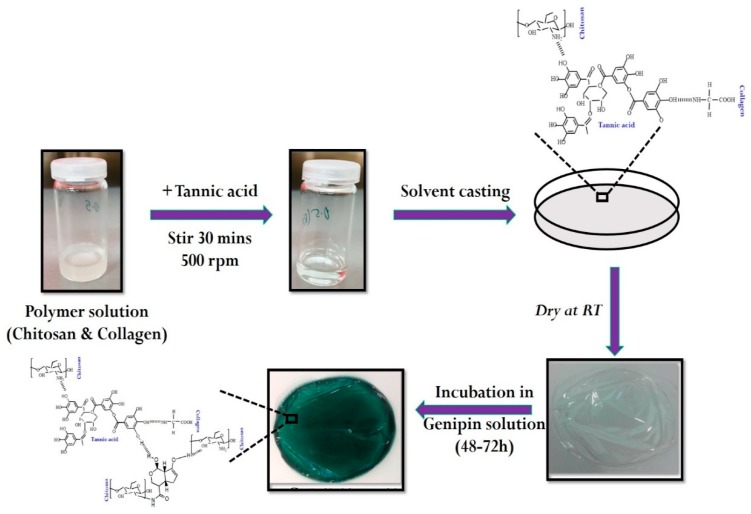
Schematic approach for the preparation of dual crosslinked collagen–chitosan films.

Dual crosslinked collagen/chitosan (Col/Chi) film was prepared through a casting technique, utilizing tannic acid and genipin as natural origin crosslinkers. To the chitosan solution (dissolved in 1% acetic acid), collagen was added in the ratio of 75:25 respectively. Then the primary cross linker, i.e., tannic acid (concentration range: 0.5%–3%, solvent: demineralized water) was slowly added portion wise into the mixture of Col/Chi. The entire mixture was stirred for 30 min at room temperature under magnetic stirring with the rotation speed of 500 rpm to ensure complete homogeneity. Thereafter, the solution is casted onto the polystyrene plates and allowed us to dry at room temperature. A smooth, transparent and flexible tannic acid crosslinked Col/Chi (Col/Chi-Ta) film was obtained. For achieving dual crosslinking in the system, the solution of genipin (concentration: 0.25%, solvent: DMSO and phosphate buffer solution) was prepared. The Col/Chi-Ta film was incubated for 48–72 h in the genipin solution for the crosslinking reaction to be achieved. Finally, the resulted genipin crosslinked Col/Chi-Ta (GpCol/Chi-Ta) film obtained had a thin, smooth texture with a greenish-blue color appearance and is termed as DC-Col/Chi (dual crosslinked collagen/chitosan) film. The entire preparation is represented in Figure 1. The blank sample was also prepared, which is devoid of any cross-linkers and comprises only of Col/Chi.

### 2.3. Characterization of Films

#### 2.3.1. Fourier Transform Infrared Spectroscopy

FTIR spectra of the films Col/Chi, Col/Chi-Ta and DC-Col/Chi were obtained at wave number of 2000–600 cm^−1^ at room temperature with uniform resolution of 4 cm^−1^ and 64 scans. For this, single beam Fourier transform infrared spectroscopy (FTIR) equipped with iD5 attenuated reflectance (ATR) was used. This ATR-FTIR was equipped with the “Omnic” software package. The crystal utilized for detecting the spectra was germanium (iD5-Ge-ATR). 

#### 2.3.2. Scanning Electron Microscopy

The morphology and porous structure of films Col/Chi, Col/Chi-Ta and DC-Col/Chi were determined by scanning electron microscopy on VEGA II LMU (TESCAN) operating at high-vacuum with an accelerating voltage 5–20 kV. The images were taken at a magnification of 100–10,000×. All the samples were sputter coated with a thin layer of palladium/gold alloy to improve the surface conductivity and tilted 30° for better observation. 

#### 2.3.3. Thermogravimetric Analysis

The TA Q500 apparatus (TA Instruments, New Castle, DE, USA) was used for thermogravimetric analysis (TGA). This analysis was performed at the constant heating rate of 10°C/min from temperature range of 25–700 °C under nitrogen atmosphere. The amount of each selected sample was approximately 10 mg.

#### 2.3.4. Swelling and Invitro Degradation Studies

Water uptake or swelling studies of the films Col/Chi, Col/Chi-Ta and DC-Col/Chi (diameter: 10 mm × 10 mm and thickness: 10 µm) were performed in phosphate buffered saline (pH 7.4; control) and in enzymatic solutions, i.e., lysozyme from chicken white (13 mg/L) and lipase from *Aspergillus oryzae* (110 U/L) at 37 °C for 2 weeks. Thereafter, time to time the swollen samples were removed from the medium and the excess water from the sample surface was removed by the filter paper. The water uptake was determined with respect to swollen samples in the control solution, i.e., PBS as well as eventual or partial degradation in the enzymatic solutions by the following equation, [34,35,36].

(1) $$\mathrm{Water}\;\mathrm{uptake}\;(\%)=\left(\frac{\mathrm{Ws}-\mathrm{Wf}}{\mathrm{Wf}}\right)\times 100$$

where, W_s_ and W_f_ are the weight of the swollen and final dry weight of test samples, respectively.

The weight loss was calculated using the equation below:

(2) $$\mathrm{Weight}\;\mathrm{loss}\;(\%)=\left(\frac{\mathrm{Wi}-\mathrm{Wf}}{\mathrm{Wf}}\right)\times 100$$

where, W_i_ and W_f_ are the initial and final dry weight of test samples respectively.

#### 2.3.5. Cell Adhesion and Proliferation Studies

The cell compatibility was detected according to previously described procedures [37]. The test samples (films Col/Chi, Col/Chi-Ta and DC-Col/Chi) were sterilized by UV radiation prior to testing. Mouse embryonic fibroblast cell line (ATCC CRL-1658 NIH/3T3; Marlboro, MA, USA) was used to test the adhesion and proliferation of cells on the surfaces. ATCC-formulated Dulbecco’s modified Eagle’s medium (Biosera, Nuaille, France) containing 10% calf serum (Biosera) and 100 U·mL^−1^ penicillin/streptomycin (PAA, Trasadingen, Switzerland) was used as the culture medium.

In the case of adhesion, the cells were seeded on the samples in the concentration of 1.106 cells mL^−1^. After one hour, the cells were stained with Hoechst 33258 (Molecular Probes, Carlsbad, CA, USA). To determine the ability of cells to proliferate on the surfaces, the cells were seeded at an initial concentration of 1.105 cells mL^−1^ and cultivated for 48 h. After 48 h the cells were fixed and stained with Hoechst 33258 and ActinRed 555 (Thermo Fisher Scientific, Waltham, MA, USA). Micrographs were taken using the fluorescence microscope Olympus IX 81 (Olympus, Tokyo, Japan).

## 3. Results and Discussion

### 3.1. FTIR Analysis of Films

**Figure 2:**
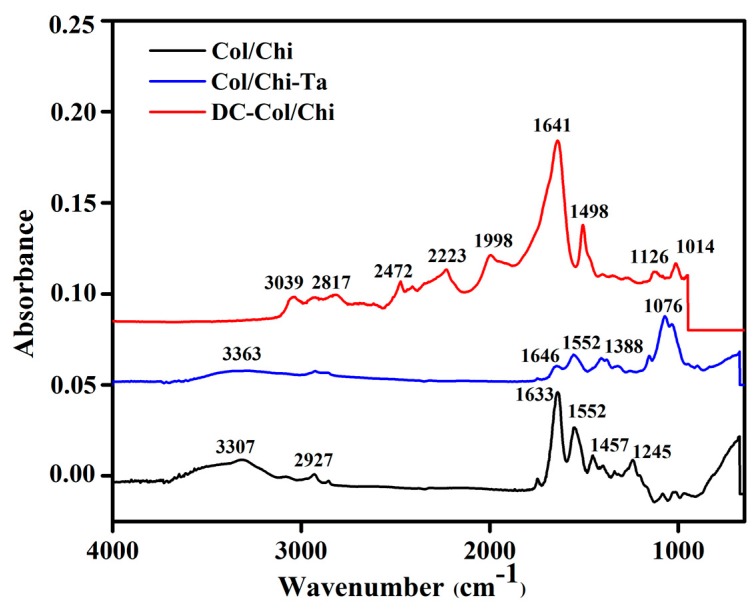
FTIR spectra of collagen/chitosan (Col/Chi), tannic acid crosslinked Col/Chi (Col/Chi-Ta) and dual crosslinked Col/Chi (DC-Col/Chi) films.

The FTIR spectra of Col/Chi, Col/Chi-Ta and DC-Col/Chi films are shown in Figure 2. In Col/Chi film spectra, the absorption bands at 3307 and 2927 represent the stretching of –OH and –CH_3_ respectively. The band at 1457 cm^−1^ is due to stretching of the pyrrolidine ring [38]. The spectrum shows the presence of collagen through the vibration band at 1245 cm^−1^ and 1552 cm^−1^ arise due to N–H bending coupled with C–N stretching vibrations indicate amide II absorbance. However, for the collagen detection, the strong signal always arises between 1700 and 1600 cm^−1^ where in the present case, it is observed at 1633 cm^−1^.

After crosslinking Col/Chi film with tannic acid spectra the shifts of bands are noticed. Tannic acid has the ability to form hydrogen bonds with the chemical moieties found in collagen and chitosan type of biopolymers. In the Col/Chi-Ta spectra, the band between around 3363 cm^−1^ represents the aliphatic –OH stretching of chitosan and tannic acid, 1646 cm^−1^ (amide I C=O stretching), 1552 cm^−1^ (amide II N–H bending and C–N stretching) of collagen and 1076 cm^−1^ for v(C–O–C) absorption [7]. Col/Chi-Ta shows new medium intensity peaks at 1388 cm^−1^ may be due to the CN stretching coupled with N–H bending [7,39].

In the crosslinking mechanism of genipin over Col/Chi-Ta, physical interaction takes place. Here there is a nucleophilic attack of the genipin C3 carbon atom with the primary amino group of the biopolymer and then embedding a tertiary N2 in the six-membered ring in place of oxygen atom [28]. The spectra of DC-Col/Chi reveals the peaks at 1641 cm^−1^, is assigned to the C=C ring stretching. These results are in accordance with the Dimida, S et al. group wherein the interaction of genipin with free amino group of polymers is shown [40]. Further, the band at 1126 cm^−1^ was assigned to the C–N stretch of the tertiary aromatic amine of genipin crosslinked with the Col/Chi-Ta. The amide II at N–H bending and C–N stretching is shifted from 1552 to 1498 cm^−1^. This is due to reaction between the genipin ester and hydroxyl groups and the amino group of chitosan within the polymeric film [41,42]. There also exists small intensity peaks between 2200 and 3000 cm^−1^, which are associated with the –OH group [43,44]. The absorption band between 1000 and 1100 cm^−1^ was attributed to C–O and C–N stretching vibrations, and C–C–N bending vibrations [41].

### 3.2. SEM Micrographs

**Figure 3:**
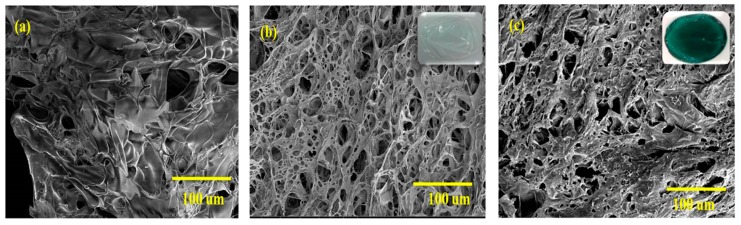
Cross section images of (**a**) Col/Chi, (**b**) Col/Chi-Ta and (**c**) DC-Col/Chi films.

Figure 3 represents the interior microstructure of crosslinked and uncrosslinked films Col/Chi, Col/Chi-Ta and DC-Col/Chi. In Figure 3a, Col/Chi matrix shows flake like structure, which is irregular in size and shape. This could be because chitosan being semi-crystal polymer tends to form the membrane structure and also there exists physical interactions of the bonds within polymers [45]. Moreover, the matrix exhibits porous nature with lack of proper alignment. After crosslinking with tannic acid and genipin consecutively significant changes were noted. The porosity of the further crosslinked Col/Chi-Ta matrix in Figure 3b was quite high even though it exhibits an irregular structure of interconnected pores. Apart from this, a honeycomb like structure was noticed with dissimilar pore sizes. However, when genipin got crosslinked to Col/Chi-Ta (Figure 3c) film, it should reveal more crosslinked structure and rehydrated, but on the contrary there was a collapse noticed in the porous structure and the fusion of the interconnected pores led to a decrease in the number of pores. 

### 3.3. TGA Analysis

**Figure 4:**
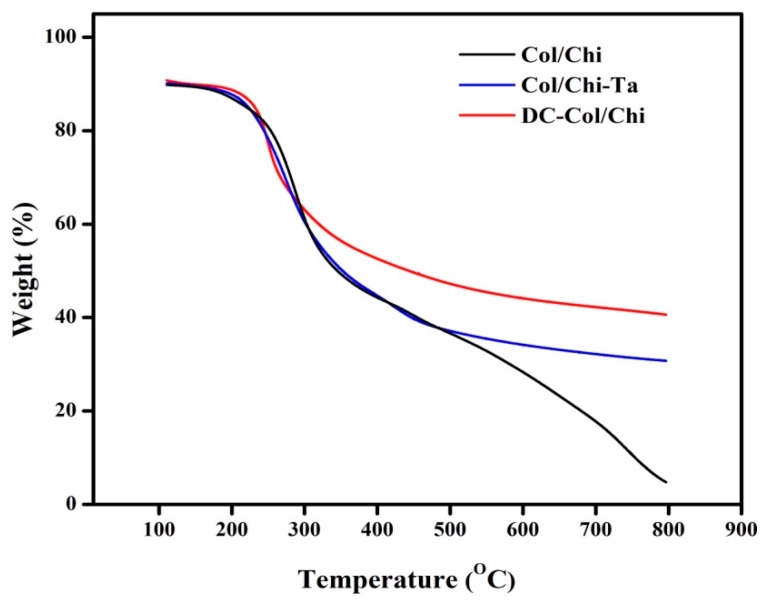
TGA of Col/Chi, Col/Chi-Ta and DC-Col/Chi films.

Thermal properties of the Col/Chi, Col/Chi -Ta and DC-Col/Chi films were studied using TGA analysis as depicted in Figure 4. Here, the initial weight loss up to 150 °C was assigned to the loss of structural bound water. The second weight loss between 300 and 340 °C was attributed to chitosan degradation. The third weight loss around 400 °C corresponded to collagen degradation, as identified by Horn et al. [46]. Usually, the weight loss until 400 °C is due to complex processes like the dehydration of the polysaccharide rings, with vaporization and removal of volatile products [41,47]. 

After crosslinking of Col/Chi with tannic acid and genipin, the polymer film exhibits a different degradation pattern. A crosslinked Col/Chi film shows water loss at the lowest heating temperature. Such findings represent the strong or weak interaction of water molecules with polysaccharides and this is clearly described by Beppu and coworkers [48]. The second stage loss between 290 and 310 °C can be due to partial decomposition of gallic acid, tannic acid or gallic acid dimers as explained by Peña and coworkers [49]. It is also interesting to know that weight loss decreases after cross linking and the stability in degradation was observed after 500 °C in both Col/Chi-Ta and DC-Col/Chi films. In DC-Col/Chi, it is clearly seen there was a significant loss after 200 °C. However, it could be concluded that the water content and thermal stability of the polymers were greatly influenced through crosslinking degree and intermolecular chain interaction.

### 3.4. Swelling and In Vitro Degradation Studies

The water uptake capacity or swelling studies of any polymeric gel/hydrogel depends on their composition, degree of crosslinking, several external conditions like temperature, pH, salt concentration, etc. The mechanism to absorb any solution (for, e.g., water, body fluids and cell nutrients) by the gel like matrixes is well explained through Donnan equilibrium theory [50]. Here, to broaden the application of the prepared films Col/Chi, Col/Chi-Ta and DC-Col/Chi, the swelling studies were performed in PBS and two different enzymes present in the human blood serum, i.e., lysozyme (also found in eyes) and lipase [36,51]. The swelling studies were carried out in the static conditions. Moreover, the swelling capacity also depends on the hydrophilicity as well as microstructure of the scaffolds. Chitosan and collagen being hydrophilic polymers, has higher water absorbing capacity [52]. So, after immersing Col/Chi film in PBS, it was impossible to assess the swelling, as it readily dissolves in the PBS solution. 

**Figure 5:**
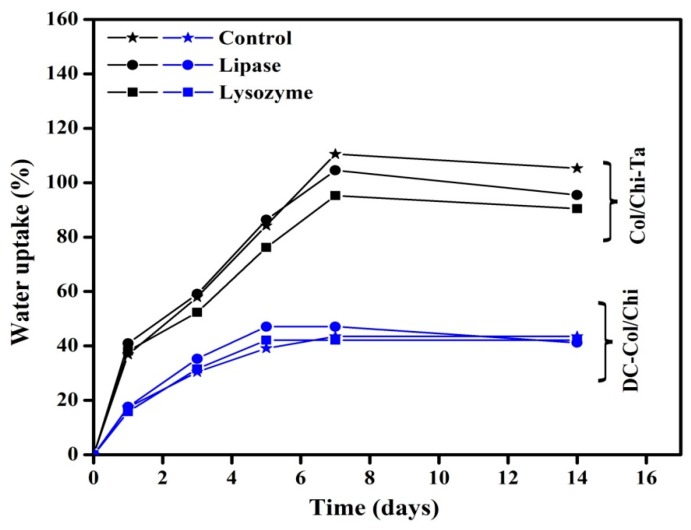
Swelling studies of Col/Chi -Ta and DC-Col/Chi films.

From the Figure 5, it is clearly visible that the swelling behavior of the Col/Chi-Ta film in PBS is significantly higher as compared to DC-Col/Chi. This could be because of the porous nature of Col/Chi-Ta, it can entrap and seize more water through capillary action. Thus the crosslinking treatment improves the scaffolds structural stability and allowing more water retention ability. However, when Col/Chi-Ta and DC-Col/Chi film is again crosslinked with genipin, there is a decrease noticed in the swelling behavior as there could be reduction in the hydrophilic groups (for, e.g., amino, hydroxyl or carboxylic groups). Moving further, the same trend (as in PBS) was observed when Col/Chi-Ta and DC-Col/Chi films were swelled in enzymatic solutions. The increasing value in the swelling of Col/Chi-Ta and DC-Col/Chi in the presence of lysozyme and lipase could be due to the degradation of the polymeric films.

When analyzing the degradation studies of Col/Chi-Ta and DC-Col/Chi films that were supplemented with lysozyme and lipase individually in PBS, the residual weight of Col/Chi-Ta and DC-Col/Chi films after degradation is depicted in Table 1. The Col/Chi-Ta film maintained 86% of its initial weight in lysozyme and 85% in lipase solution after a 14 days incubation period. The DC-Col/Chi film retained 89% and 88% of its initial weight in lysozyme and lipase solution respectively. The hydrolytic nature of the enzymes could be the cause for the degradation of the film. As a whole, both the films depict minimal degradation property reflecting positive evidence about its use as a medical implant (especially in corneal tissue engineering) and also in wound treatment.

### 3.5. Cell Adhesion and Proliferation Studies

**Figure 6:**
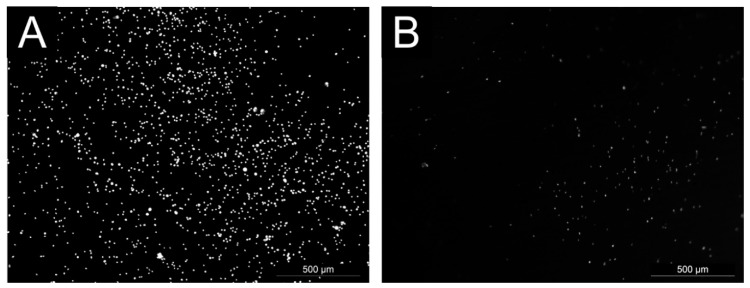
Microphotographs of cell adhesion visualized as number of cell nucleus (DNA dyed by Hoechst 33258) on reference (**A**) and DC-Col/Chi (**B**). No cell adhesion was observed on the Col/Chi and Col/Chi-Ta.

The ability of cells to adhere and proliferate on the tested surfaces, the mouse embryonic fibroblast cell line (NIH/3T3), which is one of the most frequently used lines. The cell adhesion results are shown in the Figure 6. The best cell adhesion was observed on the sample DC-Col/Chi. However, the amount of adherent cells was lower than that of the reference (tissue culture plastic). No cell adhesion was observed on the Col/Chi and Col/Chi-Ta after one hour.

**Figure 7:**
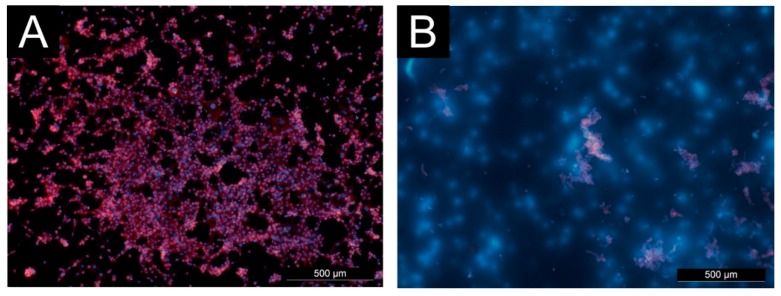
Microphotographs of cell proliferation on reference (**A**) Col/Chi-Ta No cells were observed on Col/Chi. No actin was present within the cells on (**B**) DC-Col/Chi (DNA dyed by Hoechst 33258, actin dyed by ActinRed 555).

The cell proliferation on tested surfaces is shown in the Figure 7. Due to low cell adhesion, limited cell proliferation was expected on the Col/Chi and Col/Chi-Ta films. There was no cell growth and proliferation observed on Col/Chi film, whereas on the Col/Chi-Ta film the cells were able to grow, however their proliferation was limited. It could be predicted that polycationic nature of chitosan molecules might interact with fibroblasts membrane, thus causing cell death or apoptosis. Hence, lesser the chitosan molecules within the polymeric matrix reduce the cell membrane damage [53]. Remarkable was the proliferation on the DC-Col/Chi were a number of nucleus observed, but no acting fibers were present. This is probably because the cells were actually damaged, and only a residual nucleus was present. 

## 4. Conclusions

The objective of this research work was to develop a novel dual crosslinked film that depicts a promising future in ophthalmology, skin tissue engineering and wound dressing. Firstly, Col/Chi film was prepared by a solvent casting technique and utilizing two crosslinking agents together, i.e., tannic acid and genipin. The obtained final dual crosslinked film was a translucent, thin and greenish-blue in color. The distinguishable differences among their physico-chemical properties were recorded through IR spectroscopy. The difference in the internal morphology (porosity) of the crosslinked films was visualized through SEM analysis. The thermal property was also studied using TGA analysis. Further, it was noticed that genipin crosslinked Col/Chi-Ta film exhibited lower swelling capacity. However, the degradation studies show more than 80% of the initial film weight that was retained even after 2 weeks of the incubation within the enzymatic solutions (i.e., lysozyme and lipase). Finally, the mouse fibroblasts cell adhesion and proliferation was performed indicating success in the adhesion of cells onto the genipin crosslinked matrix. However, this study definitely shows that the polymeric film constructed after the crosslinking could serve as a temporary graft in the field of ophthalmology especially for embedding over the cornea of the eyes. 

## Figures and Tables

**Table 1:** Degradation of Col/Chi-Ta and DC-Col/Chi films.

Sample Index	Degradation of Film (%)	
	Lysozyme	Lipase
Col/Chi-Ta	14	15
DC-Col/Chi	11	12
